# The Late Meeting of the American Medical Association at Nashville

**Published:** 1857-09

**Authors:** 


"*The late Meeting of the American Medical Association at Nashville.*—The meeting of this body in May last at the capital of Tennessee, presents several points for comment. In the first place, we had hoped, after what had been said by our esteemed cotemporary, the Nashville Journal of Medicine and Surgery, that the medical profession of Nashville would have declined to enter the arena for the office of President of the Association, on the ground that the practice of conferring that post upon a physician, resident at the place where the meeting is held, ought to be abolished. The reasons urged by the Nashville Journal and several similar publications, were cogent and full of point, and yet, notwithstanding this, one of the very editors of that periodical allowed himself to be nominated for the office. We mean no disrespect to the distinguished gentleman, when we say that we regret this step, because many of the opponents of the custom, which would certainly be more honored in the breach than in the observance, had reason to believe that it would have been abandoned. It is no honor to a man to be elected to office under such circumstances. It is a concession to the place; or in other words, to good eating and good cheer. We believe there is no other body of a similar kind in this country, among whom a similar custom prevails. The Moderator of the General Assembly of the Presbyterian Church, is seldom, if ever, selected from the clergy of the place where its meetings are held.

Secondly, the American Medical Profession and the American people at large, had a right to expect, after the adoption, by an almost unanimous vote, of the resolution at St. Louis, in 1854, that the custom which, up to that period, had existed, of entertaining the profession with an expensive feast, would not have been revived at the capital of Tennessee. We are told, it is true, that the Profession had no participation in the expense of the ball and supper, but that they were gotten up and paid for by the citizens of Nashville. Would it have been wrong in the Profession of Nashville to have said to their fellow-citizens, that there was a resolution upon the minutes of the proceedings of the Association, declaring that all public displays of this kind should henceforth form no part of the *transactions* of that body?

Thirdly, the meeting was a meagre one, both in respect to the number of its attendants, and in the value and importance of its transactions. Hardly any reports were presented; in almost every instance the chairmen of the different committees asked for further time. This is not as it should be. The Association, to carry out the right principles, with which it originally set out, should be more of a working and less of a pleasure-seeking body. The committees should make it their duty to discharge, with promptness and fidelity, the trust confided to them. It is a bad example to hear so many of them say that they are "not ready to report;" an example which is apt to become infectious, and to lead ultimately to the most prejudicial results. We should wake up upon this subject. To make the Association what it should be we must work more, talk less, and use a lighter diet. Moreover, the meetings should extend at least over one entire week. It is folly to think that any important business can be transacted in two or three days, by a body so large and unwieldy as this. Besides, the thing is absolutely undignified. As the matter now stands, we literally meet only to adjourn.—*N. A. Medico-Chirurgical Review.*

Upon the above we have to say, that it was our full and distinct understanding, that in the language of the journal from which the above is taken, "the medical profession of Nashville would decline to enter the arena for the office of President of the Association, on the ground that the practice of conferring that post upon a physician, resident at the place where the meeting is held, ought to be abolished."

In view especially of the declarations of the Nashville Journal, it can not be considered strange if there has been a very general disappointment (suppressed in *expression* though it has been,) in reference to this matter, in connection with which there is an *unwritten* history of "transactions" of a most remarkable character, and we cannot say in the highest possible degree creditable to all concerned.

